# Lehre im chirurgischen Tertial gestalten – Mit welchen praktischen Vorerfahrungen starten Studierende in das PJ?

**DOI:** 10.1007/s00104-025-02428-3

**Published:** 2025-12-05

**Authors:** Niklas Julian Dohle, Harm Peters, Ylva Holzhausen

**Affiliations:** Dieter Scheffner Fachzentrum für medizinische Hochschullehre und evidenzbasierte Ausbildungsforschung, Prodekanat für Studium und Lehre, Charité – Universitätsmedizin Berlin, Campus Charité Mitte, Charitéplatz 1, 10117 Berlin, Deutschland

**Keywords:** Praktisches Jahr, Chirurgie, Studentische Ausbildung, Entrustable Professional Activities, Lehre, Final year clerkship, Surgery, Undergraduate medical education, Entrustable Professional Activities, Teaching

## Abstract

**Hintergrund:**

Das chirurgische Tertial im Praktischen Jahr (PJ) soll allen Medizinstudierenden zentrale Elemente der chirurgischen Tätigkeit vermitteln. Wenig ist darüber bekannt, über welche relevanten praktischen Vorerfahrungen die Studierenden vor dem PJ bereits verfügen.

**Ziel der Arbeit:**

Ziel dieser Studie ist es, die praktischen Vorerfahrungen von Medizinstudierenden zu Beginn des PJ im chirurgischen Tertial systematisch zu erfassen.

**Material und Methoden:**

Die Vorerfahrungen wurden anhand des Konzepts der „Entrustable Professional Activities“ (EPAs) erfasst. PJ-Studierende in der Chirurgie an der Charité und deren Lehrkrankenhäusern dokumentieren ihre Vorerfahrungen in einem verpflichtenden e‑Portfolio, das auf 10 EPAs sowie 20 Prozeduren des NKLM 2.0 basiert. Analysiert wurde, wie eigenständig die EPAs und Prozeduren vor Beginn des ersten Tertials bereits durchgeführt wurden.

**Ergebnisse:**

Einbezogen wurden die Angaben von 516 Studierenden zwischen Mai 2023 und Mai 2025. Insgesamt zeigte sich eine große individuelle Variabilität in den praktischen Vorerfahrungen der Studierenden mit den unterschiedlichen EPAs und Prozeduren. Ein relevanter Anteil der Studierenden berichtete bei 7 der 10 EPAs und bei 9 der 20 Prozeduren keine bzw. geringe Vorerfahrungen.

**Diskussion:**

Die Studierenden starten mit unterschiedlichen praktischen Vorerfahrungen in das chirurgische PJ-Tertial. Eine strukturierte Erhebung der individuellen Vorerfahrungen auf Basis des EPA-Konzepts ermöglicht es, tätigkeitsbezogene Lernstände konkret sichtbar zu machen, gezielt die Zuweisung von Aufgaben und Supervision zu gestalten und individuelle Lernpläne zu entwickeln. Perspektivisch kann dies zu einer praxisnäheren chirurgischen Lehre im PJ und zu einer besseren praktischen Vorbereitung auf die ärztliche Tätigkeit beitragen und somit auch das Interesse an einer Facharztausbildung in der Chirurgie steigern.

**Graphic abstract:**

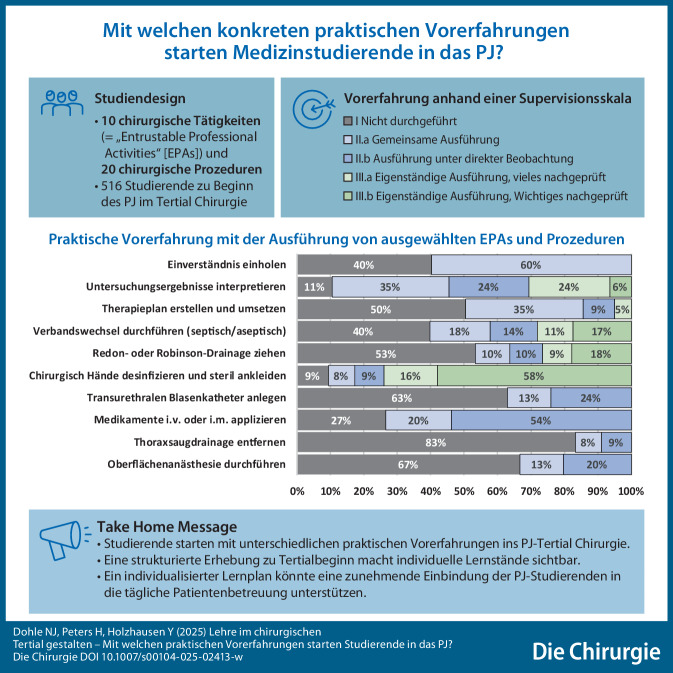

## Abstract (en)

**Background:**

The surgical rotation during the final year of medical school (practical year, PJ) is intended to equip all medical students with essential elements of surgical practice; however, little is known about the practical surgery-related experience students have prior to starting this rotation.

**Objective:**

The aim of this study is to systematically assess the practical prior experience of medical students at the beginning of the surgical rotation in the PJ.

**Material and methods:**

Prior experience was recorded using the concept of Entrustable Professional Activities (EPA). Medical students in the surgical rotation at the Charité and its teaching hospitals documented their experience in a mandatory e‑portfolio. This portfolio is based on 10 EPAs and 20 procedures defined by the National Competence-based Catalogue of Learning Objectives (NKLM 2.0). The analysis focused on the level of independence with which students had already performed these EPAs and procedures before starting their first rotation.

**Results:**

Data from 516 students between May 2023 and May 2025 were included. Overall, there was a high individual variability in students’ practical experience across the different EPAs and procedures. A substantial proportion of students reported little or no prior experience with 7 of the 10 EPAs and 9 of the 20 procedures.

**Conclusion:**

Students begin their surgical rotation in the PJ with varying levels of practical experience. A structured assessment of individual experience based on the EPA framework makes it possible to clearly identify competency levels, to tailor task assignments and supervision and develop personalized learning plans. In the long term this can contribute to more practice-oriented surgical teaching during the PJ and prepare students better for future medical practice, thereby also increasing interest in specialist training in surgery.

Die chirurgische Lehre im PJ soll zentrale ärztliche Kompetenzen vermitteln. – Doch wie starten Studierende in das PJ? Diese Studie geht der Frage nach, welche chirurgisch relevanten praktischen Vorerfahrungen PJ-Studierende zu Beginn des PJ aufweisen. Dies geschieht anhand des Konzeptes der Entrustable Professional Activities, also ärztlichen Tätigkeiten, die schrittweise an Lernende übertragen werden. Anhand des innovativen Erhebungsansatzes wird der individuelle tätigkeitsbezogene Lernstand dieser angehenden Ärztinnen und Ärzte untersucht, um Impulse für eine individualisierte Ausbildung im Tertial Chirurgie zu setzen.

## Hintergrund und Fragestellung

Die Chirurgie bildet im Praktischen Jahr (PJ) einen Grundpfeiler der praktischen Ausbildung im Medizinstudium. Mit dem verpflichtenden Tertial Chirurgie im PJ soll erreicht werden, dass alle zukünftigen Ärztinnen und Ärzte unmittelbare praktische Erfahrung mit chirurgischem Denken und Handeln in der Patientenversorgung sammeln. Um die chirurgische Lehre im PJ zu verbessern, wurden kürzlich Empfehlungen vom Berufsverband der Deutschen Chirurgie zusammen mit der Bundesvertretung der Medizinstudierenden in Deutschland im „Leitfaden Praktisches Jahr“ formuliert [10]. In diesem Leitfaden spielt das Konzept der „Entrustable Professional Activities“ (EPAs), auch „anvertraubare professionelle Tätigkeiten“ genannt, eine zentrale Rolle.

EPAs bezeichnen ärztliche Tätigkeiten, die schrittweise an Lernende übertragen werden – mit dem Ziel, dass sie diese bis zum Ende eines Ausbildungsabschnitts mit einem definierten Maß an Eigenständigkeit ausführen können [19]. International werden EPAs bereits häufig als strukturierendes Instrument in der medizinischen Aus- und Weiterbildung genutzt [3, 13, 18], auch im Bereich der chirurgischen Weiterbildung [14]. EPAs bilden eine Kernsäule des gemeinsamen Absolventenprofils des Nationalen Kompetenzbasierten Lernzielkatalogs (NKLM) 2.0 und des Gegenstandskatalogs Medizin. Hier sind konkret und fachübergreifend die EPAs aufgeführt, die Studierende im Laufe des Medizinstudiums erlernen sollten und die zur Strukturierung der Lehre im chirurgischen Tertial genutzt werden können [11].

Während des PJ sind Studierende erstmals über einen längeren Zeitraum aktiv in die Patientenversorgung eingebunden und dürfen „unter Anleitung, Aufsicht und Verantwortung des ausbildenden Arztes“ ärztliche Tätigkeiten ausführen [1]. Der Einsatz von EPAs zur Strukturierung der Lehre wird empfohlen, um sowohl den Lernenden als auch den Lehrenden eine transparente Übersicht über Lernziele sowie eine klare Grundlage für die verantwortungsvolle Aufgabenübertragung zu bieten [2, 7]. Studien zeigen zudem, dass eine aktive Einbindung in die klinische Praxis das Interesse an bestimmten Fachrichtungen fördern und die Facharztwahl beeinflussen kann – ein Aspekt, der vor dem Hintergrund des bekannten Nachwuchsproblems in der Chirurgie besondere Relevanz hat [17].

Bislang ist allerdings wenig darüber bekannt, mit welchen konkreten praktischen Vorerfahrungen Medizinstudierende ins PJ starten, u. a. da das Zweite Staatsexamen Medizin nur theoretisches Wissen abprüft. PJ-betreuende Ärztinnen und Ärzte haben somit keine ausreichend klare Vorstellung davon, welche praktischen Fähigkeiten sie von den Studierenden im chirurgischen Tertial erwarten können. Ein besseres Verständnis dieser Ausgangslage könnte dazu beitragen, die Lehre im PJ gezielter zu gestalten und die Studierenden effektiver in eine zunehmend eigenständige, supervidierte Patientenversorgung einzubinden. Ziel dieser Studie ist es, die praktischen Vorerfahrungen von Medizinstudierenden zu Beginn des PJ in ihrem chirurgischen Tertial systematisch zu erfassen.

## Studiendesign und Untersuchungsmethoden

### Studiendesign

Die wiederholte Querschnittstudie wurde an der Charité – Universitätsmedizin Berlin (Charité) durchgeführt. Grundlage der Erhebung waren Angaben aus einem EPA-basierten elektronischen Portfolio (e-Portfolio), das im Mai 2023 eingeführt wurde. Das e‑Portfolio ist u. a. von allen Studierenden verpflichtend zu führen, die ihr chirurgisches PJ-Tertial an der Charité oder einem der 44 Lehrkrankenhäuser absolvieren. Das e‑Portfolio an der Charité folgt dem Konzept eines Logbuchs, in dem die Studierenden ihre praktischen Erfahrungen dokumentieren. Ziel des e‑Portfolios ist es zum einen, den Studierenden und den PJ-betreuenden Ärztinnen und Ärzten eine Übersicht und Vorgabe über die im PJ erwarteten Tätigkeiten und Supervisions- bzw. Eigenständigkeitsgrade zu geben. Zum anderen erhalten die PJ-Studierenden ein strukturiertes, formatives Feedback zu ihrer Ausführung einzelner Tätigkeiten.

Die Analyse der im e‑Portfolio dokumentierten Daten, wie in dieser Studie vorgenommen, erfolgte mit Zustimmung der zuständigen Ethik-Kommission (EA1/212/23) auf Basis eines umfassenden Datenschutzkonzeptes. Sie erfolgte im Einklang mit nationalem Recht sowie gemäß der Deklaration von Helsinki von 1975 (in der aktuellen, überarbeiteten Fassung).

### Erfassung der praktischen Vorerfahrung

Die praktischen Vorerfahrungen der PJ-Studierenden wurden auf Basis des EPA-Konzepts erfasst, d. h. als definierte ärztliche Tätigkeiten und als operationalisierte Supervisions- bzw. Eigenständigkeitsgrade.

**Abb. 1:**
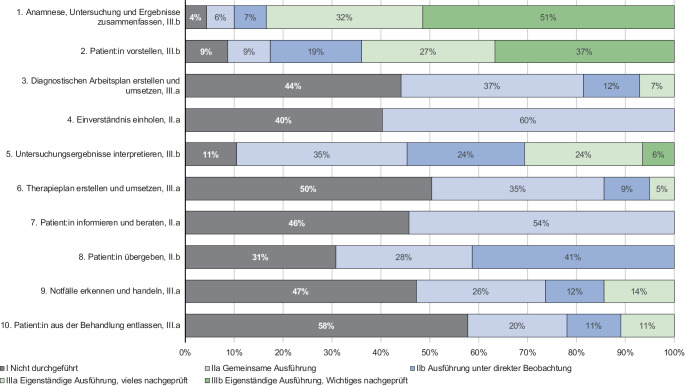
Praktische Vorerfahrung mit der Ausführung von Entrustbale Professional Activities vor Beginn des PJ

Die im e‑Portfolio abgebildeten ärztlichen Tätigkeiten basieren auf 10 EPAs, die im Absolventenprofil des NKLM 2.0 definiert sind [11]. Ergänzend wurden für die EPA „Ärztliche Prozeduren durchführen“ insgesamt 20 ärztliche Prozeduren in Zusammenarbeit mit chirurgischen Fachvertreterinnen und -vertretern der PJ-Kommission spezifiziert, die für das chirurgische Tertial relevant sind. Eine Übersicht der Titel der EPAs und Prozeduren findet sich in Abb. 1 und 2 im Ergebnisteil. Eine detaillierte Beschreibung der EPAs und Prozeduren mit Titel, Spezifikation, Limitation, Supervisionsgrad am Ende des PJs sowie Kenntnissen, Fertigkeiten und Haltungen ist im EPA Booklet der Charité zu finden (siehe Infobox Weiterführende Literatur).

**Abb. 2:**
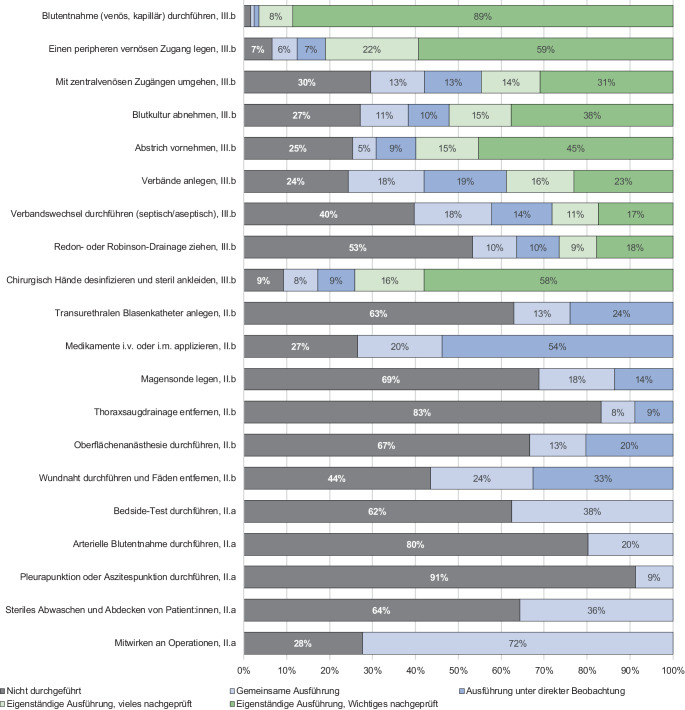
Praktische Vorerfahrung mit der Ausführung von Prozeduren vor Beginn des PJ

Der Grad an Eigenständigkeit bei der Ausführung einer Tätigkeit wurde in Anlehnung an die definierten Supervisionslevel von Chen et al. anhand einer 6‑stufigen Skala operationalisiert (Tab. 1; [6]).

**Tab. 1:** Skala zur Erfassung der praktischen Vorerfahrung durch Eigenständigkeitsgrade

	Eigenständigkeitsgrade
0.	Weder durchgeführt noch beobachtet
I.	Beobachten/demonstriert bekommen
II.a	Gemeinsame Ausführung mit Ärztin/Arzt
II.b	Ausführung durch Student:in unter direkter Beobachtung einer Ärztin/eines Arztes
III.a	Eigenständige Ausführung, vieles musste nachgeprüft werden
III.b	Eigenständige Ausführung, nur Wichtiges musste nachgeprüft werden

### Datenerhebung

Die Daten wurden von Mai 2023 bis Mai 2025 unter insgesamt 5 Studierendenkohorten erhoben. Eingeschlossen wurden nur Daten von Studierenden, die ihr PJ mit dem chirurgischen Tertial begonnen haben. Im Rahmen des e‑Portfolios wurden diese in den ersten beiden Tertialwochen aufgefordert, für alle EPAs und Prozeduren zu dokumentieren, ob, und wenn ja, mit welchem Eigenständigkeitsgrad sie diese mindestens 3‑mal ausgeführt haben. Die Originalformulierung der entsprechenden Frage lautete: *„Ich habe die EPA oder Prozedur hinreichend sicher und effektiv unter folgendem Supervisionslevel ausgeführt … (höchster erreichter Supervisionsgrad bei mindestens 3‑facher Ausführung).“* Die anonymisierten Erhebungsdaten wurden im Mai 2025 aus der Datenbank für die Auswertung exportiert.

### Datenanalyse

Die statistische Auswertung erfolgte mit IBM SPSS Statistics, Version 29.0.0.0 (241) (IBM Corporation, Armonk, New York, USA). Für jede EPA und Prozedur wurde der prozentuale Anteil der Studierenden ermittelt, die die jeweilige Tätigkeit auf den einzelnen Stufen des Eigenständigkeitsgrads ausgeführt haben. Zur Vereinfachung der Darstellung wurden die Stufen 0 („Tätigkeit weder durchgeführt noch beobachtet“) und I („Beobachten/demonstriert bekommen“) zusammengefasst. Ein Eigenständigkeitslevel von 0/I zeigt an, dass die betreffende EPA oder Prozedur von der bzw. dem Studierenden noch nicht selbst ausgeführt wurde.

## Ergebnisse

Von den insgesamt 533 eingeschlossenen Studierenden füllten 516 (97 %) die Erhebung zu den praktischen Vorerfahrungen am Tertialbeginn vollständig aus. Davon absolvierten 31 % ihr Tertial an den chirurgischen Kliniken der Charité, während 69 % an einem der akademischen Lehrkrankenhäuser der Charité tätig waren; 69 % der Befragten gaben die Charité als ihre Heimatuniversität an, 31 % kamen von anderen Universitäten. Bezüglich der gewählten chirurgischen Fachdisziplinen entschieden sich die meisten Studierenden für die Allgemein- und Viszeralchirurgie (53 %) und die Unfallchirurgie (28 %).

### Praktische Vorerfahrungen mit EPAs

Die Analyse der Vorerfahrungen zeigt Unterschiede zwischen den Studierenden hinsichtlich der Tätigkeiten, die sie bereits vor Beginn des PJ ausführten, und dem Grad der Eigenständigkeit, unter dem sie die einzelnen EPAs ausführten (s. Abb. 1). In der Gesamtgruppe finden sich größere Vorerfahrungen bei der EPA 1 „Anamnese erheben, untersuchen und Ergebnisse zusammenfassen“ und der EPA 2 „Patient:in vorstellen“. Hier haben jeweils 51 % und 37 % der Studierenden angegeben, diese Tätigkeiten bereits unter dem Eigenständigkeitsgrad III.b („nur Wichtiges muss nachgeprüft werden“) ausgeführt zu haben.

Bei 7 der 10 EPAs gab jedoch rund jede:r dritte Studierende an, bislang keine praktischen Vorerfahrungen gesammelt zu haben. So hatten beispielsweise 44 % der Studierenden keine praktische Vorerfahrung mit der EPA 3 „Diagnostischen Arbeitsplan erstellen und umsetzen“ und 46 % mit der EPA 7 „Patient:in informieren und beraten“. Jede:r zweite Studierende gab an, noch keine praktische Erfahrung mit der EPA 6 „Therapieplan erstellen und umsetzen“ (50 %) und der EPA 10 „Patient:in aus der Behandlung entlassen“ (58 %) zu haben.

### Praktische Vorerfahrungen mit Prozeduren

Die Analyse der Vorerfahrungen mit den 20 Prozeduren zeigt ein vergleichbares Bild wie bei den EPAs, nämlich deutliche Unterschiede zwischen den Studierenden hinsichtlich der Tätigkeiten, die sie bereits vor Beginn des PJ ausführten, und dem Grad der Eigenständigkeit bei der Ausführung (s. Abb. 2). In der Gesamtgruppe finden sich größere Vorerfahrungen bei den 3 Prozeduren „Blutentnahme durchführen“, „Einen peripheren venösen Zugang legen“ und „Chirurgisch Hände desinfizieren und steril ankleiden“. Auch hier gab rund jede:r zweite Studierende an, diese Tätigkeiten bereits unter Eigenständigkeitsgrad III.b („Nur Wichtiges muss nachgeprüft werden“) ausgeführt zu haben. Bei 9 von den 20 Prozeduren gab hingegen mehr als jede:r zweite der Studierenden an, noch keinerlei praktische Erfahrung mit der Ausführung zu haben. Beispielsweise gaben 80 % der Studierenden an, noch keine arterielle Blutentnahme durchgeführt, eine Thoraxdrainage entfernt (83 %) oder eine Pleura- und Aszitespunktion durchgeführt zu haben (91 %). Dabei zeigt sich die Tendenz, dass insbesondere geringe Vorerfahrung bei Prozeduren besteht, die von den Studierenden am Ende des PJ entweder gemeinsam oder unter direkter Aufsicht eines Arztes oder einer Ärztin durchgeführt werden sollten.

## Diskussion

Im PJ-Tertial Chirurgie sollen angehende Ärztinnen und Ärzte grundlegende Erfahrungen im chirurgischen Denken und Handeln bei der Patientenversorgung sammeln. Der NKLM und auch die Empfehlungen vom Berufsverband der Deutschen Chirurgie zusammen mit der Bundesvertretung der Medizinstudierenden in Deutschland sehen vor, die Lehre im PJ anhand von EPAs zu strukturieren, sodass Studierende Schritt für Schritt auf die eigenständige Ausführung von essenziellen chirurgischen Tätigkeiten vorbereitet werden [10, 11]. Im Rahmen dieser Untersuchung wurden mittels einer umfassenden, wiederholten Querschnittstudie erstmals die praktischen Vorerfahrungen von Medizinstudierenden mit der Ausführung von EPAs und Prozeduren zu Beginn des Praktischen Jahrs im Chirurgie-Tertial analysiert. Die Ergebnisse zeigen, dass eine große individuelle Variabilität in der praktischen Vorerfahrung mit diesen definierten ärztlichen Tätigkeiten vorliegt und dass ein beachtlicher Teil der Studierenden bei einer Reihe von Tätigkeiten keinerlei Vorerfahrung aufweist. Im Folgenden werden der methodische Ansatz, die gefundenen Ergebnisse und die möglichen Implikationen dieser Studie für die chirurgische Lehre im PJ diskutiert.

Methodisch-konzeptionell nutzte diese Studie das EPA-Konzept, ein national im NKLM 2.0 verankertes und international etabliertes Instrument zur strukturierten Erfassung der klinisch-praktischen Kompetenzen der Studierenden [11]. Durch die Auswahl von 10 EPAs sowie 20 Chirurgie-relevanten Prozeduren und die Operationalisierung der Eigenständigkeitsgrade war eine differenzierte und standardisierte Analyse des Ausbildungsstands zu Beginn des PJ-Tertials möglich. Die Umsetzung der Erhebung in einem verpflichtenden e‑Portfolio trug erheblich zur Datenqualität und einer Rücklaufquote von 97 % bei. Der Anteil von 31 % Studierenden, die ihr Medizinstudium an anderen Fakultäten absolviert haben, unterstreicht die Bedeutung der Ergebnisse über den Standort Charité hinaus [12].

Bei den Ergebnissen der Erhebung ist insbesondere die ausgeprägte Variabilität in den praktischen Vorerfahrungen der Studierenden zu Beginn des chirurgischen PJ-Tertials bemerkenswert. Diese breite Streuung deckt sich mit den Ergebnissen bisheriger Studien (beispielsweise [9, 16]). Besonders auffällig ist die nicht vorhandene praktische Erfahrung jedes dritten Studierenden mit 7 EPAs, die zu den grundlegenden ärztlichen Tätigkeiten gehören. Zudem gab über die Hälfte der Studierenden bei 9 von 20 definierten chirurgischen Prozeduren an, keinerlei praktische Vorerfahrung zu haben. Insgesamt zeigen die Ergebnisse eine Individualität der praktischen Vorerfahrungen der Studierenden und spiegeln die unterschiedlichen Ausbildungswege wider.

Die Ergebnisse dieser Studie haben mögliche Implikationen für die Lehre im Tertial Chirurgie sowie für das Medizinstudium insgesamt. Die Individualität der praktischen Vorerfahrungen der einzelnen Studierenden unterstreicht die Notwendigkeit, diese zu Beginn eines jeden PJ-Tertials bei den Studierenden auf Basis des EPA-Konzepts mit definierten Tätigkeiten und operationalisierten Eigenständigkeitsgraden zu erheben. Dies kann bei Nichtvorhandensein eines e‑Portfolios oder e‑Logbuchs auch papierbasiert erfolgen. Den PJ-betreuenden Ärztinnen und Ärzten bietet dies eine fassbare Grundlage, um den PJ-Studierenden gezielt Aufgaben in der Patientenversorgung zu übertragen. Dabei können die PJ-betreuenden Ärztinnen und Ärzte individuell für jede EPA oder Prozedur entscheiden, inwieweit sie auf den angegebenen Vorerfahrungen aufbauen wollen oder ob sie die Studierenden zu Beginn enger supervidieren wollen. Die Erhebung des Ausgangsniveaus der oder des einzelnen Studierenden bildet gleichzeitig die Grundlage für die Entwicklung eines individuellen Lernplans, d. h. es wird konkret identifiziert, wo Lücken bestehen, wie diese im aktuellen Arbeitsbereich geschlossen werden können, welcher Eigenständigkeitsgrad erreicht werden soll und welche Lernmomente und Feedbackschwerpunkte hierfür notwendig sind [4]. Am Ende des PJ-Tertials bietet das Konzept den PJ-betreuenden Ärztinnen und Ärzten schließlich die Möglichkeit, mit ihren PJ-Studierenden individuell zu reflektieren, inwieweit die Ziele für die Studierenden im PJ bei der Ausführung der Tätigkeiten erreicht wurden. Dies könnte insgesamt zu einer von den Studierenden eingeforderten besseren Betreuungsqualität durch Lehrende, der Möglichkeit zur eigenständigen Patientenbehandlung sowie der Vorbereitung auf die berufliche Praxis führen [8].

### Limitationen

Bei der Interpretation der Studienergebnisse sind einige Limitationen zu berücksichtigen. Erstens basiert die Erhebung auf Angaben der Studierenden, was subjektive Verzerrungen und unterschiedliche Interpretationen der Eigenständigkeitsgrade nicht ausschließt. Zweitens wurden nicht alle im NKLM 2.0 definierten EPAs und Prozeduren erfasst – die Auswahl beschränkte sich auf 10 EPAs sowie 20 chirurgisch relevante Prozeduren, die lokal in Abstimmung mit Fachvertreter:innen definiert wurden. Diese Auswahl schränkt die Übertragbarkeit auf alle EPAs des NKLM ein. Drittens handelt es sich bei der Stichprobe mehrheitlich um Studierende der Charité. Zwar kamen 31 % der Studierenden von anderen Universitäten, dennoch könnte das Ergebnis durch curriculare Besonderheiten oder eine selektive Wahl des chirurgischen Tertials an der Charité verzerrt sein. Diese Limitationen relativieren die Reichweite der Befunde, mindern jedoch nicht deren Bedeutung für die gezielte Weiterentwicklung einer EPA-basierten chirurgischen Lehre im PJ.

### Ausblick

EPA-basierte (elektronische) Logbücher ermöglichen eine strukturierte Erfassung praktischer Vorerfahrungen und könnten individuelle Lernprozesse im PJ fördern [15]. Dies könnte die Qualität der Supervision verbessern, eigenständiges Arbeiten gezielt unterstützen und das Interesse an der Chirurgie stärken. Zukünftige Studien sollten hierzu evaluieren, ob durch den Einsatz EPA-orientierter Formate tatsächlich ein Zugewinn praktischer Kompetenzen erreicht wird und sich ein gesteigertes Interesse an einer chirurgischen Facharztausbildung ableiten lässt. Perspektivisch sollten EPA-orientierte Formate zudem bereits im Studium verankert und für die Weiterbildung anschlussfähig gestaltet werden, um einen kontinuierlichen Kompetenzaufbau von der Ausbildung bis zur Facharztreife zu ermöglichen [5].

#### Infobox Weiterführende Literatur

Das Handbuch „Entrustable Professional Activities – Für das Praktische Jahr – In den Fächern Innere Medizin, Chirurgie, Anästhesiologie“ ist aufrufbar unter https://dsfz.charite.de/lernen_und_lehren.

## Fazit für die Praxis

- Studierende bringen sehr unterschiedliche praktische Vorerfahrungen mit ins PJ-Tertial Chirurgie.
- Eine strukturierte Erhebung zu Tertialbeginn (z. B. mit einem EPA-basierten Logbuch oder Portfolio) macht individuelle Lernstände sichtbar.
- Darauf aufbauend können PJ-betreuende Ärztinnen und Ärzte die Zuweisung von Aufgaben und Supervision an PJ-Studierende gezielt anpassen.
- Ein individualisierter Lernplan könnte eine zunehmende Einbindung der PJ-Studierenden in die tägliche Patientenbetreuung unterstützen und die eigenständige Arbeit der PJ-Studierenden stärken.
- So könnte die PJ-Lehre praxisnäher gestaltet und das Interesse an einer Facharztausbildung in der Chirurgie potenziell gesteigert werden.

## Data Availability

Die erhobenen Datensätze können auf begründete Anfrage in anonymisierter Form beim korrespondierenden Autor angefordert werden. Die Daten befinden sich auf einem Datenspeicher am Dieter Scheffner Fachzentrum an der Charité-Universitätsmedizin Berlin.
